# Evaluation of repositioning care provided by non-professionals using a caregiver-assistive device: an experimental study

**DOI:** 10.1038/s41598-023-48377-x

**Published:** 2023-11-30

**Authors:** Yuka Omura, Atsuko Watanabe, Kasumi Shibata, Tomoko Inoue

**Affiliations:** 1 Graduate School of Medical Safety Management, Jikei University of Health Care Sciences, Osaka, Japan; 2 Division of Health Sciences, Osaka University Graduate School of Medicine, Osaka, Japan; 3 Global Center for Medical Engineering and Informatics, Osaka University, Osaka, Japan; 4 Faculty of Nursing, Morinomiya University of Medical Sciences, Osaka, Japan; 5 Faculty of Nursing, Osaka Medical and Pharmaceutical University, Osaka, Japan

**Keywords:** Occupational health, Geriatrics, Health occupations

## Abstract

As the population ages in Japan and worldwide, the number of informal caregivers, such as family members, providing nursing care to older individuals is increasing. Among caregiving tasks, repositioning care, which causes lower back pain, is frequent and burdensome for caregivers. Therefore, we developed a position-changing device that can adjust and support the care recipient’s body in the lateral position. This was a feasibility study of the device-assisted care provided by non-professionals using the device we developed. Of the 40 healthy volunteers enrolled, 17 simulated caregivers and 17 simulated care recipients finally participated in the study. One caregiver and one care recipient were paired to engage in two types of care: device-assisted care and manual care. Furthermore, the care provided by the caregiver and received by the care recipient were evaluated. Non-professionals were able to use the device successfully and safely after a short period of practice, and both caregivers and care recipients rated the device-assisted care positively. The study results suggest that informal caregivers can also provide safe and comfortable care that is less burdensome than manual care by using a caregiver-assistive device.

## Introduction

In 2021, 28.9% of Japan’s population was aged 65 years or older, making Japan one of the most aged countries in the world^1^. The proportion of the older adults is expected to exceed 30% of the total population by 2025^2^. In response to this aging population, Japan has established community-based integrated care systems that provide housing, medical care, nursing care, preventive care, and lifestyle support in a unified framework that enables people to continue to live in familiar communities until the end of their lives, even if they become severely in need of nursing care, and has promoted the use of home and community-based nursing and long-term care services^3^. The number of family members responsible for caregiving has increased; the number of family caregivers aged 15 years and older in Japan in 2021 was 6,534,000^4^. Aging is a major problem not only in Japan but also in many European countries, such as Italy and Germany, and in some Asian countries, such as South Korea^1, 5^. In developed countries, the number of informal caregivers is increasing as the number of people requiring nursing care is increasing, and the shortage of caregivers has become a problem^6–8^. In low- and middle-income countries, the number of people requiring nursing care is increasing, owing to the rise in lifestyle-related diseases, although limited access to healthcare has resulted in care being provided by informal caregivers^9–11^.

Informal caregivers play a major role in improving the quality of life of those in need of care, preventing facility placement and reducing the length of hospitalization^12^; however, the poor health of the caregiver can affect the quality of care provided^13^. Thus, it is important to maintain the health of informal caregivers to maintain the continuity of care.

The burden of caregiving can lead to the development of low back pain (LBP) in informal caregivers, who lack professional training or education in caregiving^10, 14, 15^. They often provide care alone because it is difficult to obtain support from other persons^14, 16^ and face difficulties related to accessing equipment at home as opposed to that at a facility^14^ and the limited care space in the home environment^14, 16^, all of which contribute to the high risk of LBP. Through a questionnaire survey of 156 female family caregivers in Japan, Suzuki et al. found that caregiving activities related to LBP included night-time care and repositioning^15^. Repositioning care is required during various care activities, such as preventing pressure ulcers, changing sheets, observing wounds, providing hygiene care, and inserting lift slings. Position changes are physically demanding and cause occupational health problems, including LBP, even for professionals with specialized knowledge, skills, and training^17^.

In addition, inexperienced caregivers show behavior patterns that place them at a higher risk of developing LBP than experienced caregivers^18^. Although the prevalence of LBP among informal caregivers is not as clear as that among nurses and other professionals, 76%^14^ and 64.7%^9^ of informal caregivers have been reported to show back symptoms. Furthermore, Suzuki et al. reported that 80% of family caregivers complained of LBP and 20% experienced LBP daily^15^, indicating the severity of the situation.

Sliding sheets are used to reduce the physical burden of caregivers during repositioning, and their usefulness has been demonstrated among nurses^19^. However, among informal caregivers, the use of sliding sheets did not decrease the rating perspective exertion (RPE), suggesting that their use may not fully benefit informal caregivers^20^.

We developed a novel caregiver-assistive device that can be used during bedridden care; professional caregivers and nurses have demonstrated the effectiveness of the device in reducing caregiver burden^21^. This study aimed to evaluate the feasibility of using this device in home care situations by informal caregivers.

## Methods

### Key features of the device

**Figure 1:**
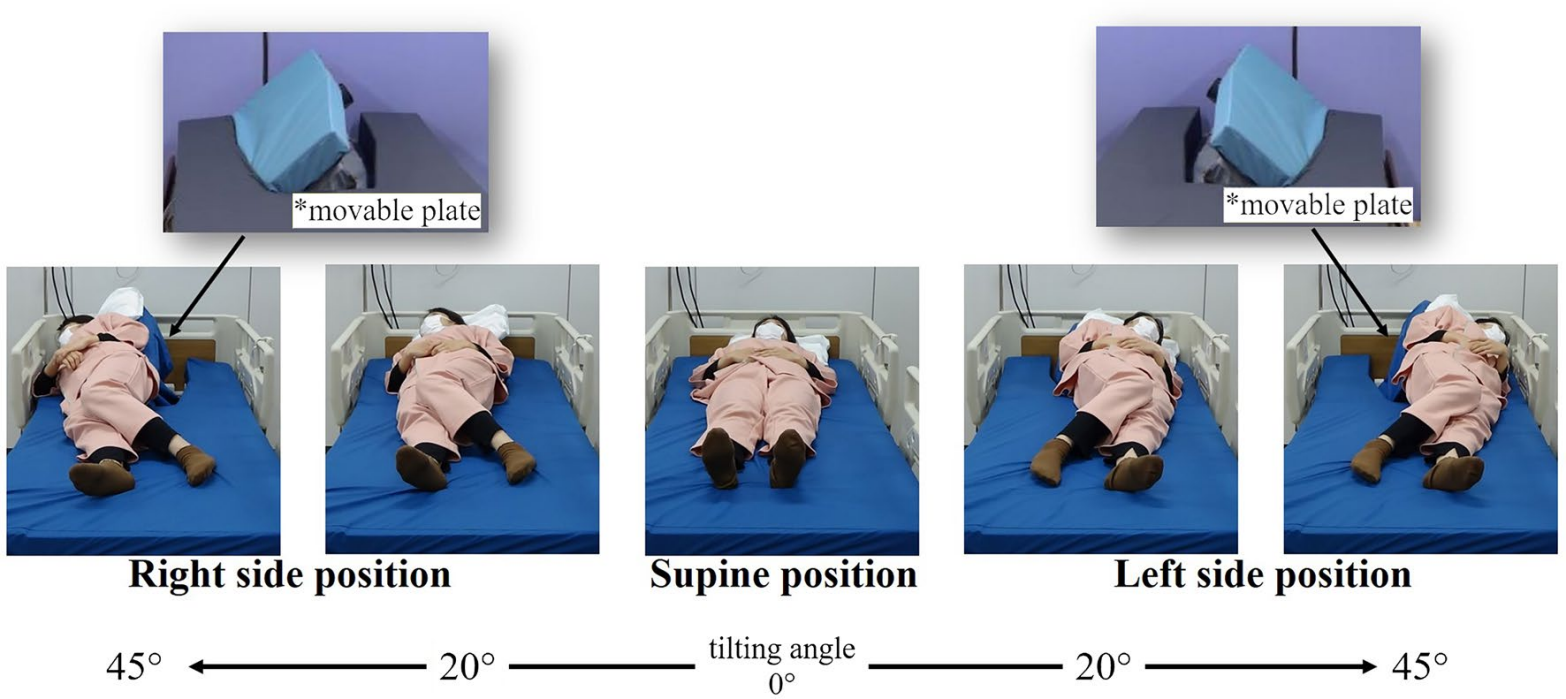
Positions enabled by the device. Raising a part of the mattress allows the device to tilt the care recipient’s body laterally and support the care recipient’s body when providing care. The tilt angle is controlled in the range of 0° to 60°, and caregivers can easily operate the system by pressing buttons on the remote control. When the tilting button is pressed once, it tilts at a constant speed up to the pre-set angle. For example, the angle can be set to 45° during nursing care (diaper changing) and 20° during rest time. When the supine mode button is pressed once, the movable plate (*photos are reprinted from Omura et al.^21^) moves into the horizontal position; thus, the care recipient’s body is in the supine position^21^.

The device used in this study was developed by our group^21^ and has two main features: it can raise a part of the mattress to tilt the care recipient’s body laterally and can support the care recipient’s body when providing care (Fig. 1). These features eliminate the caregiver’s physical workload during lateral tilting. The device can tilt and support the care recipient’s body automatically; therefore, the caregiver can provide care with both hands, and the help of another caregiver to stabilize the body tilt during care is not required. This feature reduces the mental burden of performing multiple tasks involving physical labor while observing the care recipient’s health conditions during the care. Furthermore, compared with the manual method, this device can ensure care recipient comfort by offering stable support and gentle tilting. In addition, it has customizable operating speeds and tilt angles. The use of this device does not require special medical qualifications. As it adopts a similar method to that of traditional electric beds, where adjustments can be made with a remote control, it is considered a machine usable by anyone who can use traditional beds.

### Participants

In this study, informal caregivers were defined as individuals with varying degrees of caregiving experience, but without formal, specialized education in caregiving, possessing only limited knowledge and skills related to care. They differ from professional caregivers, who have received specialized education in caregiving, possess extensive and adequate knowledge and skills related to caregiving, and have the experience to provide safe and comfortable care. Female nursing students enrolled in Bachelor’s degree nursing program who had taken basic nursing technique courses to acquire fundamental knowledge of biomechanics skills to prevent LBP were selected. In Japan’s Bachelor’s degree nursing programs, nursing students at graduation are considered capable of performing basic nursing assistance techniques under instructional guidance^22^. Notably, the practice of nursing care is rarely conducted independently by students, and practical experience primarily occurs after obtaining a national nursing license and entering clinical settings. Therefore, Japanese nursing students have limited knowledge, skills, and experience, making them equivalent to informal caregivers. Furthermore, all the nursing students who participated in this study had no prior experience in activities such as repositioning care recipients outside of their educational settings. The researcher, who was not directly involved in the education of nursing students, recruited the participants in February 2022 via verbal discussions and flyers that were sent to students who had completed the basic nursing skills course. Those who had LBP that interfered with their lives or had health problems that were deemed unsuitable for study participation by the researcher were excluded.

The care recipients were healthy female individuals interested in nursing and caregiving who understood the study and who played the role of a care recipient. In addition, the weight of the care recipients was limited to maximum 62 kg (calculated from the average height of female individuals aged 20–29 years, 158.7 cm)^23^ and body mass index < 25 kg/m^2^ (the upper limit of normal body weight) because the heavier the care recipient, the greater the physical burden on the caregiver. The volunteer care recipients were recruited through verbal discussions and flyers at the facility in February 2022.

### Study design

**Figure 2:**
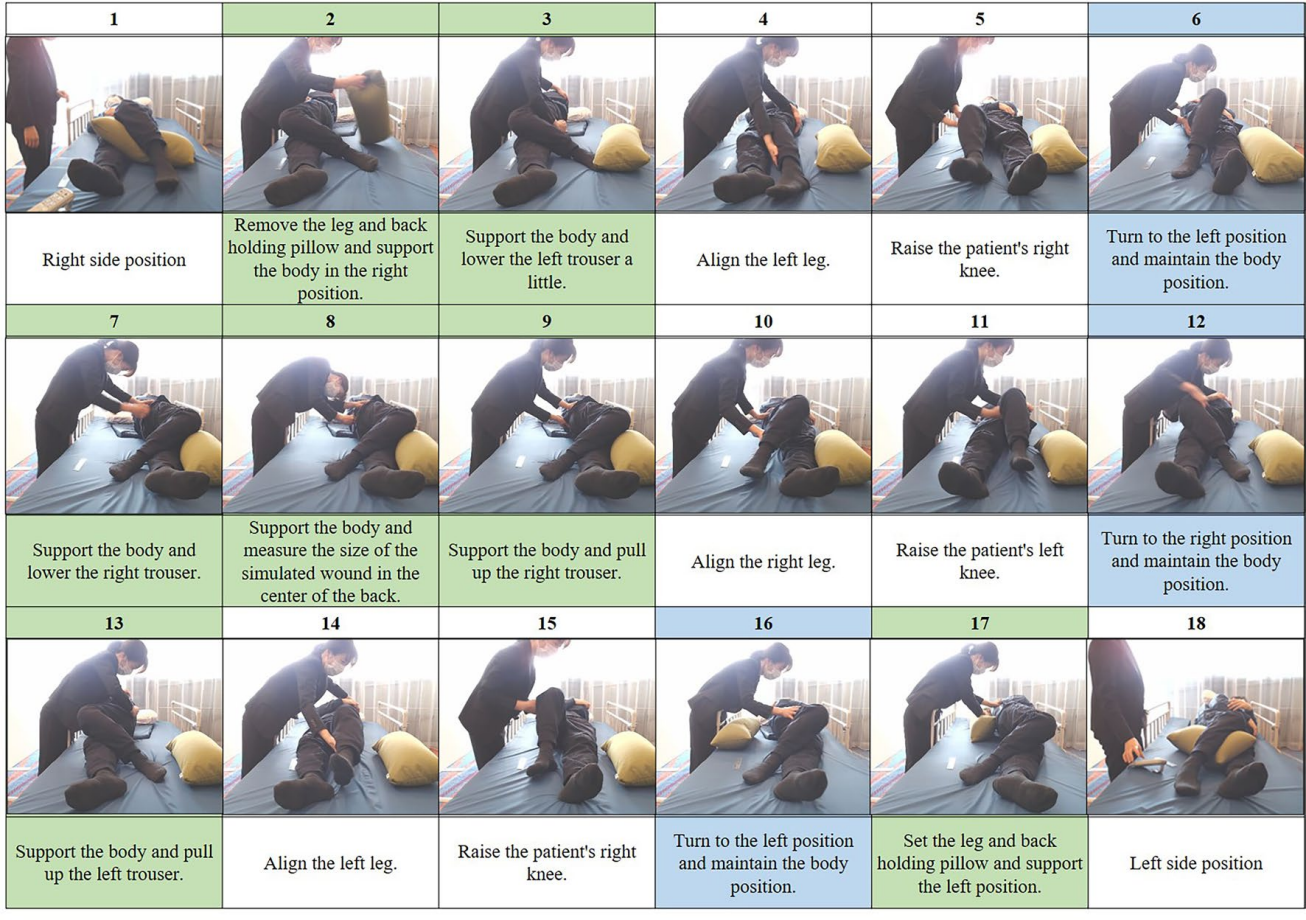
Repositioning care task. In the repositioning task, the wound care procedure was simulated with the care recipient lying on the right side of the bed, and the care recipient’s position was changed to the left side. Blue items (6, 12, 16) denote the parts of device-assisted care where the device replaces the repositioning tasks, and green items (2, 3, 7, 8, 9, 13, 17) denote the parts of device-assisted care where the device supports the care recipient’s body. White items (1, 4, 5, 10, 11, 14, 15, 18) denote the same procedures in device and manual care.

This was a comparative study using an experimental design. The outcomes of two care methods were compared: device-assisted care by one caregiver (device-assisted method) and care without the device by one caregiver (manual method). One caregiver (hereafter referred to as “caregiver”) was paired with one volunteer care recipient (hereafter referred to as “care recipient”). The caregiver performed a simulated wound care procedure on the care recipient who was lying on the right side of the bed and changed the care recipient’s position to the left (hereafter referred to as “repositioning care task”) (Fig. 2) with both methods (device-assisted method and manual method).

**Figure 3:**
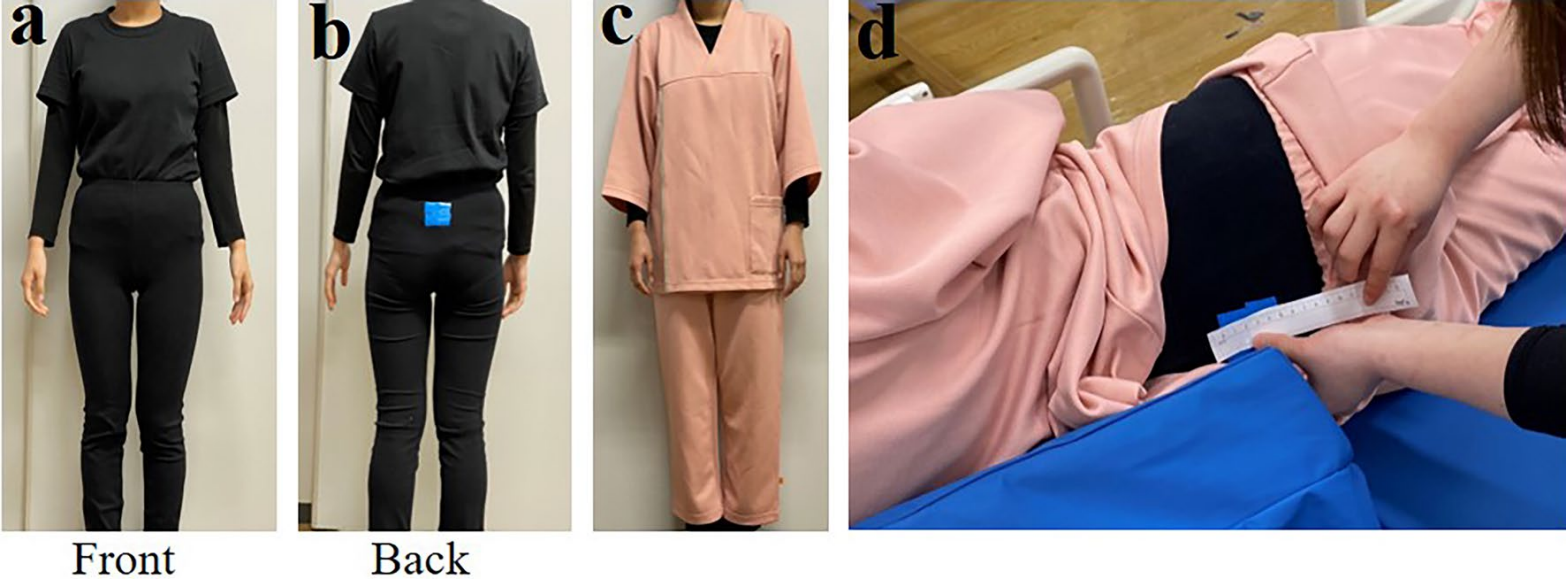
Details of simulated wound care. Each care recipient wore a T-shirt and leggings/tights (**a**) and pajamas over the underwear (**c**). The simulated wound was patched on the tights (**b**). In the lateral lying position, the caregiver pulled down the pajama pants, observed the wound, and measured its size using a scale (**d**).

The “simulated wound care” procedure involved the caregiver placing the care recipient in the lateral position, pulling down the pajama trouser, observing the wound, and measuring its size with a scale (Fig. 3). The simulated wound was placed on the lower back (near the fourth lumbar vertebra [L4], affixed on the line connecting the right and left iliac crests) with a 3-cm square blue cloth tape.

**Figure 4:**
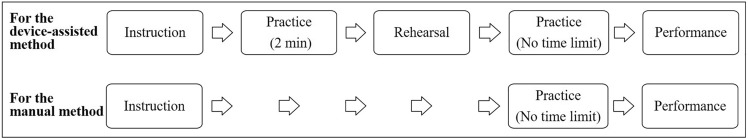
Flow of experiments for device-assisted care and manual care. For the device-assisted method, after receiving an explanation of the device and care instructions, the caregivers practiced caregiving using the device for 2 min with a dummy and then performed the device-assisted method once on the volunteer (rehearsal). The caregiver then practiced with the dummy until she was confident that she could perform the care smoothly. Then, she performed the procedure using the device-assisted method with the care recipient (performance). For the manual method, after receiving an explanation of the manual care procedure, the caregivers practiced manual care with a dummy until they were confident that they could perform the care procedure smoothly; they then performed it using the manual method once (performance), without 2 min of practice and rehearsal.

As shown in Fig. 4, after receiving an explanation of the device and care instructions, each caregiver practiced the care scenario using the device for 2 min with a dummy [Practice] and then performed the procedure using the device-assisted method once on the care recipient [Rehearsal]. The caregiver then practiced with the dummy until they felt that they could perform the care procedure smoothly and performed it using the device-assisted method a second time [Performance]. Next, after receiving an explanation of the manual care procedure, the caregiver practiced manual care with a dummy until they judged that they could perform the care smoothly [Practice]; then, they performed the manual method once [Performance]. All participants performed the procedure in the same sequence, starting with the device-assisted method, followed by the manual method.

**Figure 5:**
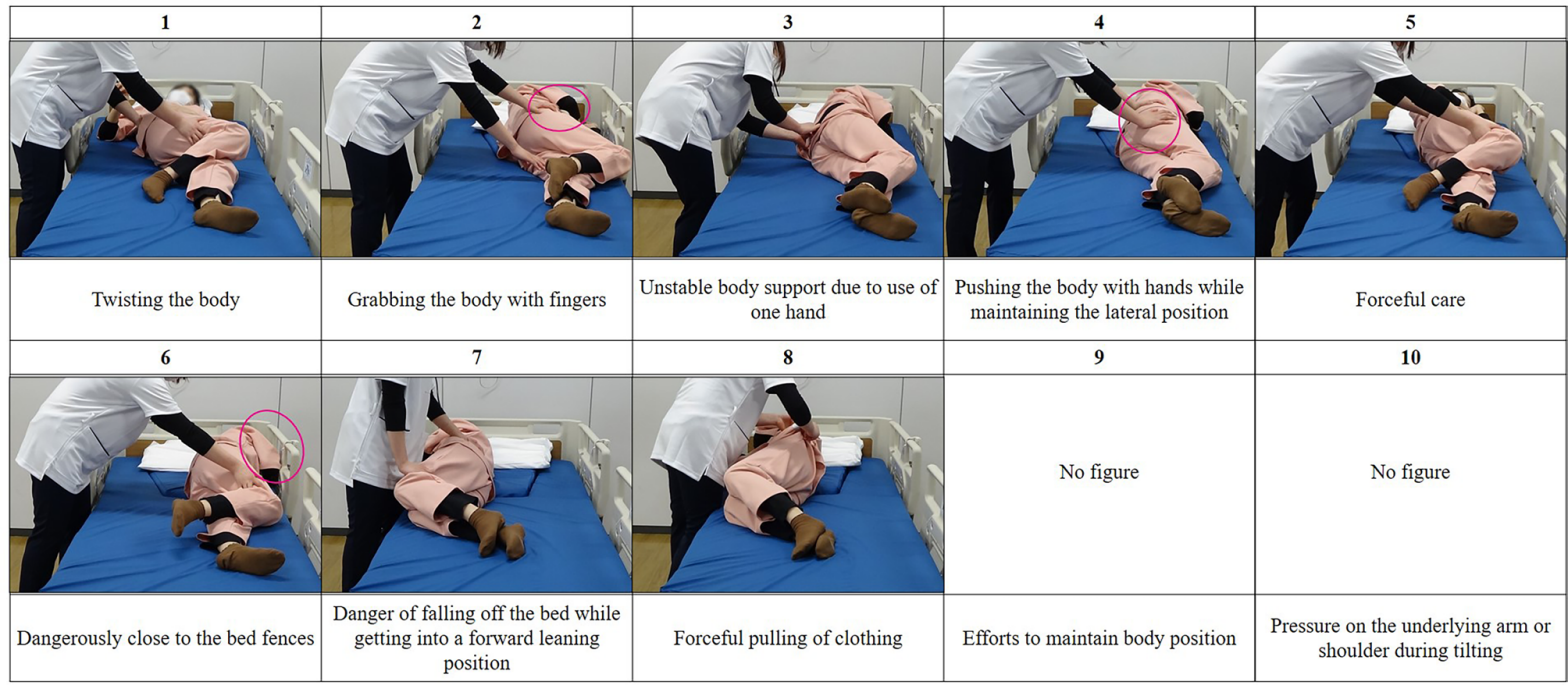
The 10 perspectives for the evaluation of care.

The care recipients were instructed by the researcher on how to act as care recipients based on 10 perspectives of care evaluation with an illustration (Fig. 5): “1. My body was twisted by the caregiver (Twisting the body)”, “2. My body was grabbed with the caregiver’s fingers (Grabbing the body with fingers)”, “3. I felt unstable body support due to use of only one caregiver’s hand (Unstable body support due to use of one hand)”, “4. My body was pushed with the caregiver’s hands while I was in a lateral position (Pushing the body with hands while maintaining lateral position)”, “5. I felt that the care provided by the caregiver was brute-force care (Forceful care)”, “6. I felt in danger as I was close to the bed fences (Dangerously close to the bed fences)”, “7. I leaned forward and felt like I was in danger of falling off the bed (Danger of falling off the bed while getting into a forward leaning position)”, “8. I felt the caregiver forcefully pulling my clothing (Forceful pulling of clothing)”, “9. I tried to maintain my body position (Efforts to maintain body position)”, and “10. My arms and shoulders were pressed under my body during tilting (Pressure on the underlying arm or shoulder during tilting)”. Our previous study related to care recipients^24^ revealed that a sense of bodily twist, body posture stability, and forced care were associated with the satisfaction of care. As a result of this research, items 1 to 5, 8, and 9 were developed. In addition, a separate study among caregivers involved in device-based care^21^ showed that caregivers expressed concerns regarding approaching bed fences, the risk of falls from the bed, and the impact on the limbs of individuals with contractures. Therefore, in this study, items 6, 7, and 10 were added to gather the perspectives of care recipients too. After the care recipient changed into the proper clothes, an electrocardiographic heart rate sensor (Wireless biological monitor, RF-ECG2, GMS Co., Ltd, H41-W44-D9.7 mm; weight, 15 g) was attached using two electrocardiogram electrode patches (Blue sensor, P-00-S, METS Inc., Japan, Diameter 34 mm) on the left anterior chest by the researcher. The care recipient lay on the bed in a resting position with closed eyes.

### Setting

The experiments were conducted in the university’s nursing practice room at the researchers’ facility in Osaka, Japan, in March 2022. The device consists of a mattress and an underlying tilting unit attached to the bed frame with fixtures. The bed frame used in this study was equipped with an electric switch that raised the bed height, and the left and right bed fences could be unlocked by pulling out. The width of the mattress on the bed frame was 83 cm. The participants acted as the dependent care recipients. To ensure that each volunteer accurately represented a bedridden care recipient with communication difficulties and no limb movement, they were instructed to provide no assistance to caregivers and relax their muscles. In addition, the researcher explained the mechanism and features of the device to the care recipient beforehand and confirmed that the care recipient acted as a bedridden care recipient by lying on the device.

### Outcomes

#### Primary outcome:

##### Subjective care assessments

At the end of each task, subjective care assessments were performed from three perspectives in line with the fundamental concepts of nursing care^25–27^: safety (the absence of danger), comfort (the state of feeling calm), and ease (care is implemented smoothly and without delay). Correspondingly, the three concepts were assessed by the caregivers and care recipients using the following questions: Were you able to provide care safely?/Was the care you were provided safe?, Were you able to provide care comfortably?/Was the care you were provided comfortable?, and Were you able to provide care smoothly and without delay?/Was the care you were provided smooth and without delay?. A 100-point visual analog scale was used, with 0 points indicating no safety and 100 points indicating strong safety. They were also asked to rate their care scores on a 100-point scale. In addition to these ratings, the care recipients were also asked to rate specific 10 indicators according to the care recipients’ perspectives mentioned earlier on a 6-point Likert scale (1: very often, 2: often, 3: a little, 4: few, 5: merely, 6: not at all) at the end of each task.

#### Secondary outcomes:

##### Care time and practice time

Care time was defined as the time (in s) from the beginning to the end of the task, and practice time was defined as the time (in s) required to practice the task with the dummy until the caregiver judged that they could perform the care smoothly (no time limit practice).

##### Rating perspective exertion (RPE)

The Borg Scale for Perceived Exertion was used to evaluate the caregivers’ subjective assessment of the physical exertion required to complete the task of turning and positioning the care recipient in the bed. This instrument uses a scale ranging from 6 (no exertion) to 20 (maximum exertion)^28, 29^. The reliability and validity of the Borg Scale have been previously reported^28^. The Borg Scale has been used in a previous study^30^. The RPE was measured using a questionnaire administered to caregivers after each task.

##### Occurrence of adverse events

The occurrence of adverse events, such as pinching of the mattress, care recipient bumping into the bed fences or falling off the bed due to mishandling of the device, and care recipient feeling discomfort caused by the device, was checked.

##### Heart rate of care recipients

As an objective indicator of the physical burden on the care recipients, the heart rate of the care recipients during care was measured using an electrocardiographic heart rate sensor. This study focused on intra-individual differences of the heart rate. The sensor was maintained from the beginning of the study until the end of the final care session. Data were extracted using a specific software (MemCalc/Bonaly Light; GMS Co., Ltd, Japan) and analyzed for each care session.

### Statistical analyses

Paired t-tests were performed to compare the caregivers’ and care recipients’ ratings of care, RPE of caregivers, and heart rate of care recipients between the device-assisted and manual care. JMP® was used for data analysis, with a significance level of 5%. The 10 indicators according to the care recipients’ perspectives were evaluated using a 6-point Likert scale divided into two categories: Yes, the event has occurred (the sum of [very often], [often], and [a little]) and No, the event has not occurred (the sum of [little], [merely], and [not at all]).

Sample size calculations using pretest data were not conducted; however, reference was made to many previous studies regarding evaluations for the developed device or evaluations by care recipients. Similar to the current study, a study by Nodooshan et al.^30^ evaluated a device from the point of view of the care recipient, with four respondents as care recipients and 37 respondents as caregivers^30^. In studies of device evaluations by caregivers, Omura et al. included 27 and 28 participants in their studies^19, 21^. Hwang et al. included 20 caregivers to evaluate the devices^31^, while Pay et al.’s study on device evaluation by non-expert caregivers had 20 participants^20^. Thus, based on previous studies in this research area, the sample size was set at 20 pairs.

### Registration number and name of the study registry

This study was registered in the University Hospital Medical Information Network (registration number: UMIN000046651). All methods were performed according to relevant guidelines and regulations.

### Ethical considerations

All participants provided oral and written informed consent prior to the experiment, and the experimental protocol was approved by the Osaka University Clinical Research Review Committee (approval number: 21357).

## Results

### Participant information

Twenty pairs of caregivers and care recipients were recruited. However, two caregivers and one caregiver and care recipient pair were absent; thus, these three pairs were excluded from the analysis. The characteristics of the caregivers and care recipients are listed in Table 1.

**Table 1:** Characteristics of participants.

Variable	Care givers (n = 17)			Care recipients (n = 17)		
	Value			Value		
	Mean	SD	Range	Mean	SD	Range
Age (years)	21.6	0.9	20–23	19.4	0.6	19–21
Body weight (kg)	50.9	5.9	40–63	49.4	3.0	45–55
Height (cm)	158.9	5.3	150–169	158.2	4.9	150–166

*SD* standard deviation.

### Care assessments by caregivers

As shown in Table 2, caregivers rated the device-assisted care as safer (diff =  − 20.6, p = 0.0007), more comfortable (diff =  − 32.6, p < 0.0001), and easier (diff =  − 24.4, p = 0.0012) than manual care, and the device-assisted care score (diff =  − 19.7, p = 0.0006) was significantly higher. The caregivers’ RPE, as per the Borg scale scores^23^, was also significantly lower for device-assisted care by 5.3 points (p < 0.0001).

**Table 2:** Mean and differences between the device-assisted and manual methods using a paired t test (caregivers, n = 17).

Dependent variables	Score range/unit	D		M		Diff	SE	95% CI		t-value	p-value
		Mean	SD	Mean	SD			Lower	Upper		
Safety	0–100	85.6	13.1	65.0	18.2	− 20.6	5.0	− 10.1	− 31.2	− 4.16	**0.0007**
Comfort	0–100	83.5	12.3	50.9	21.1	− 32.6	5.0	− 22.0	− 43.2	− 6.51	** < 0.0001**
Ease	0–100	82.4	22.1	58.0	20.8	− 24.4	6.2	− 11.3	− 37.4	− 3.95	**0.0012**
Care score	0–100	80.9	14.0	61.2	19.5	− 19.7	4.7	− 9.8	− 29.6	− 4.23	**0.0006**
RPE	6–20	8.8	2.6	14.1	2.9	5.3	0.8	6.9	3.7	6.91	** < 0.0001**
Care time	seconds	136.4	18.6	127.7	24.3	− 8.7	4.2	0.1	− 17.5	− 2.09	0.0529
Practice time	seconds	203.6	127.9	119.5	78.6	− 84.2	21.7	− 38.2	− 130.1	− 3.88	**0.0013**

*D* device-assisted method; Diff: differences, *M* manual method, *SE* standard error, *RPE* rating perceived exertion measured by the Borg’s scale. Score 7 is called “Very, very light”, Score 9 is called “Very light”, Score 11 is called “Fairy light”, Score 13 is called “Somewhat hard”, Score 15 is called “Hard”, Score 17 is called “Very hard”, and Score 19 is called “Very, very hard”.

Significant values are in bold.

### Care assessments by care recipients

**Figure 6:**
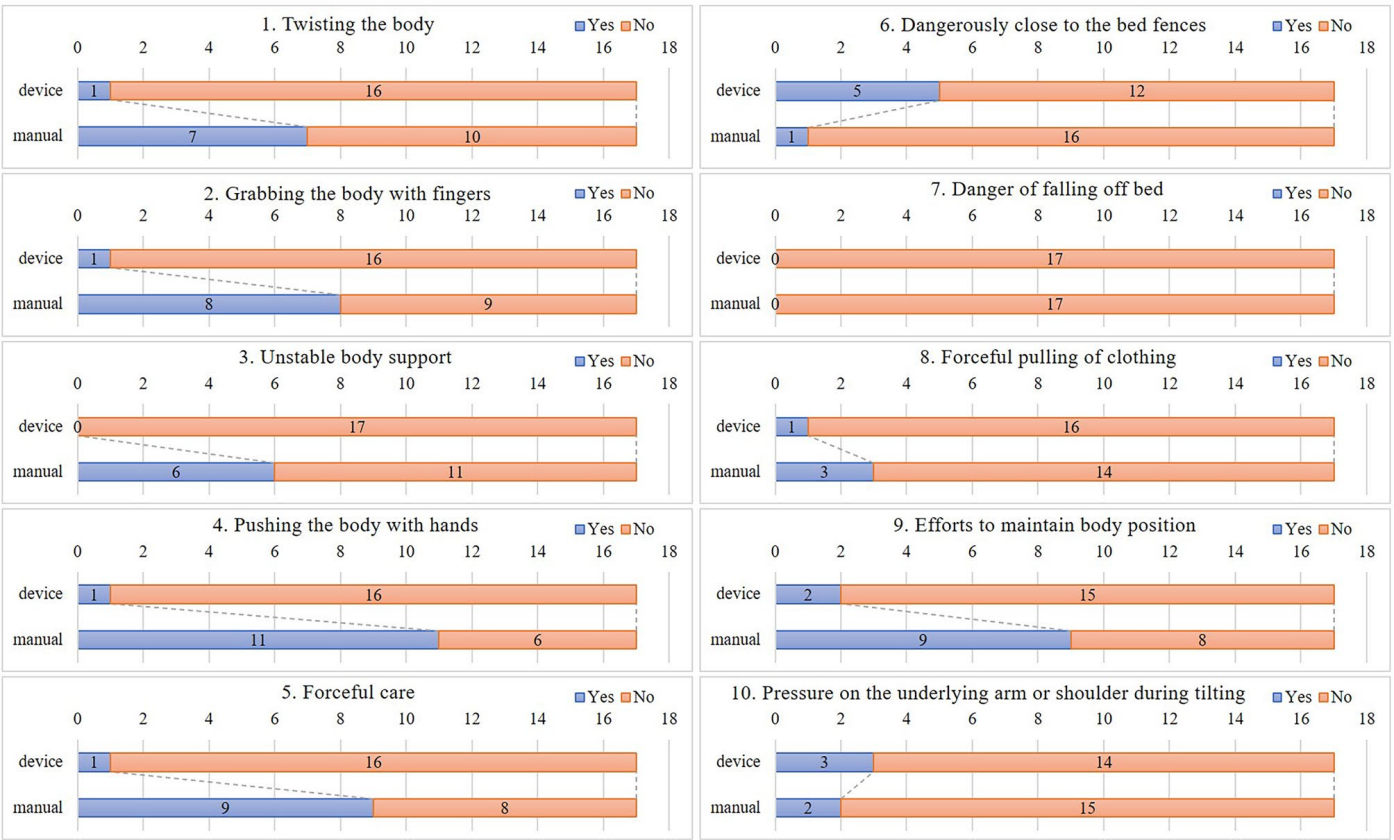
Care evaluation based on the 10 indicators according to care recipients’ perspectives (n = 17). The blue bar indicates that the event has occurred, the sum of [very often] [often], and [a little] whereas the red bar indicates that the event has not occurred, the sum of [little], [merely], and [not at all].

As shown in Table 3, care recipients also rated device-assisted care higher than manual care in terms of safety (diff =  − 13.1, p = 0.0119), comfort (diff =  − 21.5, p = 0.0016), and ease (diff =  − 26.0, p = 0.0002); the device-assisted care score (diff =  − 18.4, p = 0.0011) was also significantly higher. The heart rate of care recipients was not significantly different between the two methods (diff = 0.9, p = 0.2802). The 10 perspectives of care were evaluated (Fig. 5). Of these, “1. Twisting the body”, “2. Grabbing the body with fingers”, “3. Unstable body support”, “4. Pushing the body with hands”, “5. Forceful care”, “8. Forceful pulling of clothing”, and “9. Efforts to maintain body position” were lower for device-assisted care (Fig. 6). In contrast, “6. Dangerously close to the fences” and “10. Pressure on the underlying arm or shoulder during tilting” were more frequently reported for device-assisted care. “7. The danger of falling out of bed” was not reported for either care type.

**Table 3:** Mean and differences between the device-assisted and manual methods using a paired t test (care recipients, n = 17).

Dependent variables	Score range/unit	D		M		Diff	SE	95% CI		t-value	p-value
		Mean	SD	Mean	SD			Lower	Upper		
Safety	0–100	90.3	9.3	77.2	20.6	− 13.1	4.6	− 3.3	− 22.8	− 2.84	**0.0119**
Comfort	0–100	91.6	8.1	70.2	24.2	− 21.5	5.7	− 9.4	− 33.5	− 3.78	**0.0016**
Ease	0–100	92.2	6.6	66.2	21.6	− 26.0	5.3	− 14.7	− 37.3	− 4.88	**0.0002**
Care score	0–100	90.9	5.3	72.6	18.8	− 18.4	4.6	− 8.6	− 28.1	− 3.99	**0.0011**
Heart rate	Beat/min	61.1	9.9	61.9	9.6	0.9	0.8	2.5	− 0.8	1.12	0.2802

*D* device-assisted method, *Diff* differences, *M* manual method, *SE* standard error.

Significant values are in bold.

### Care time and practice time

As shown in Table 2, there was no significant difference in the mean care time spent between the device-assisted and manual care groups (diff =  − 8.7, p = 0.0529). The mean practice time for device-assisted care was 203.6 s. The mean practice time for manual care was 119.5 s. There was a significant difference in the mean practice time spent between the device-assisted and manual care groups (diff =  − 84.2, p = 0.0013).

### Occurrence of adverse events

All 17 pairs completed the care tasks as scheduled without requiring changes in the protocol or experiencing adverse events, such as pinching of the mattress, bumping into the bed fences, falling out of bed owing to incorrect use of the device, or feeling ill because of repositioning care.

## Discussion

This study aimed to evaluate the feasibility of a caregiver-assistive device and evaluations of device-assisted care. In our experimental setting, healthy (non-professional) participants were asked to perform a simulated task of back wound treatment involving lateral position changing.

First, the caregivers found that using the device enabled them to provide care to the care recipient more comfortably and safely, and they assigned care scores nearly 20 points higher than those of the manual method. Furthermore, the RPE score, which indicates the caregiver’s perceived burden, decreased by 5.3 points with the use of this device. RPE is measured using a 15-point scale, with scores ranging from 6 to 20. In a study evaluating a patient transfer device between bed and stretcher^30^, the score for the manual method was 18 points, which reduced to 8 points when using the device. However, in the present study, the score for the manual method was 14.1 and reduced to 8.8 on device use. The relatively small change of 5.3 points might be because the present study focused on repositioning care, involving somewhat less physical exertion than transfers. Nonetheless, the use of the device effectively reduced the score to 8.8 points, which signifies “very light” to “very very light” physical burden.

Second, care recipients evaluated the device-assisted care as being safe and comfortable, and their care scores were also high. In particular, “Twisting the body”, “Grabbing the body with fingers”, “Unstable body support”, “Pushing the body with hands”, “Forceful care”, and “Efforts to maintain body position”, which were linked to the comfort aspect of care, reduced in frequency with device use. The use of this device enables informal caregivers to achieve the device’s specific functions of “Slow lateral position repositioning” and “Stable support to the care recipient’s body”. Professional caregivers possess the knowledge and skill of how to place their hands comfortably and apply gentle force when changing the position of a care recipient^32^. They ensure a stable transition without causing any anxiety or concerns, ultimately creating a sense of safety. By using the assistive device, even informal caregivers can potentially guarantee comfort and safety to care recipients.

In addition, the heart rate did not differ significantly between those who used and did not use the device, suggesting no effect on the care recipients’ cardiovascular system. However, the use of the device increased concerns related to proximity to the bed rails and a sensation of pressure on the arms. These concerns were also raised by professional caregivers in a previous study^21^. While we expected this device to contribute to the comfort of care recipients through gentle repositioning, it appears to have created a sensation of the bed rails encroaching gradually, as well as gradual pressure on the arms and shoulders. Additionally, in the previous study^21^, use of the device ensured less body slippage than with manual repositioning. Thus, it is essential to conduct thorough risk assessments, such as measuring the actual distance to the rails. Furthermore, specific attention should be given to informal caregivers, as they might become overly engrossed in providing care, possibly leading to their faces being close to the bed rails or failing to notice pressure on the arms. Therefore, there is a need to assess and implement awareness measures for informal caregivers when using the device.

Third, the practice time with the device had a standard deviation of 2 min, with a maximum of 7 min. On average, the participants took approximately 4 min to master the operation of the device. This device only requires pressing buttons to control the tilting to the left and right, and it was assumed that the presence or absence of medical knowledge would not have a significant impact, making it suitable for informal caregivers. Of note, the practice time for the device method was 84 s longer than that for the manual method, posing a challenge in terms of the increased effort required to master the device method. In the future, further improvements such as more forthright instructions and interfaces that allow intuitive use will be essential to reduce the time needed to become proficient with the device. However, given that the participants were informal caregivers, not all could perform repositioning care. Since the study involved a practice protocol encompassing both the use of the device and care technique itself, the extended practice time might be attributed to the dual focus on learning how to use the device and care technique. Informal caregivers are at high risk of LBP due to little professional training and education in caregiving^10, 14, 15^. The results of the current study suggest that this device, which can be mastered in a short practice time, may be beneficial for such informal caregivers at high risk of LBP.

In summary, the repositioning device proved valuable to informal caregivers, as it could be used with minimal practice time and simple guidance and without adverse events. This device showed the potential to reduce physical strain on caregivers and enhance safety, comfort, and ease of care for both caregivers and care recipients.

With the rise in home care, the number of families unfamiliar with caring for older individuals is expected to increase rapidly. Providing effective support to caregivers^13^ and improving social support and caregiver competence^33^ have been shown to improve the quality of care. We believe that engineering-assisted care methods will improve the care provided by informal caregivers.

## Strengths and limitations

This study focused on repositioning care, which is a burdensome task for caregivers, and verified the comfort of the care recipients and reduction in caregivers’ burden. It was found that even non-professionals can provide comfortable care for the care recipients with this device. However, this study has the following limitations.

First, healthy young women were recruited as informal caregivers and bedridden care recipients. With the rise in the aging population, long-term caregiving has become a common phenomenon, and in Japan, 63.5% of primary caregivers are ≥ 65 years old and 35.7% are ≥ 75 years old^34^. Therefore, there may be a decline in the ability to memorize and learn device operation, as well as hand dexterity, when operating remote-controlled devices among older caregivers. In this regard, further data collection from informal older caregivers is necessary. In addition, the characteristics of bedridden care recipients, such as restricted limb movements and difficulty communicating, differ from those of the volunteer care recipients in the present study.

Additionally, despite evaluating caregiver assistive device intended for use among older adults, the selection of younger individuals as care recipients limits the generalizability of the findings to older care recipients. For example, older adults often experience chronic pain^35^ and a decline in motor function^36^, which can lead to pain when moved or repositioned involuntarily. Therefore, if bedridden older individuals were the care recipients, the comfort and care scores may have been lower than those reported herein. The cutoff weight for the care recipients in this study was 62 kg or less; however, the maximum weight observed was 55 kg. Thus, the device’s capacity, which allows a weight of up to 100 kg has not yet been verified. For future implementation, it is necessary to expand the target population to include a wider range of ages and body types.

Second, all participants performed the device-assisted method first, followed by manual care, and the order of interventions was not randomly assigned. Therefore, the possibility of order effect cannot be ruled out. As care recipients received care more times, they may have become familiar with the flow of care, allowing them to feel more at ease during care. As a result, their evaluations of care might have become more sensitive and nuanced, and the manual care evaluations performed later were possibly more critical. Similarly, caregivers providing care may have gained proficiency not only in operating the device but also in the repositioning care itself through repetition and practice. Consequently, the care evaluations, particularly in terms of smoothness, might have progressively improved. Future studies should consider randomized controlled trials, where the order of manual and device care is assigned randomly.

Third, in this study, the evaluations of care were subjective and did not include muscle activity or postural evaluation, which can biometrically measure the physical burden of the caregiver. However, it has also been shown that inexperienced caregivers exhibit behavior patterns that are associated with a higher risk of developing LBP than experienced caregivers^14^, and as a next step, not only subjective aspects of the informal caregiver’s use of the device but also postural/biomechanical evaluation should be conducted.

Fourth, this study was conducted in an experimental setting, not a home environment; thus, the possibility that the clinical situation was not accurately reproduced cannot be dismissed. In a home environment, things are not always in good condition, such as scattered items, and these aspects place an increased burden on the home caregiver. However, the equipment is built into the bed, eliminating the need to set up a sling, as is the case with lifts; we believe that this feature can be beneficial. When using a care lift, there is additional work such as positioning a sling beneath the individual. In contrast, this device has a built-in mattress on the bed, eliminating the need for extra preparations like laying a sling. However, before use (before the care recipient lies down), this device needs to be pre-installed on the bed frame. Furthermore, in addition to the standard bed controller, a separate controller is required, necessitating an extra power outlet. Therefore, using the device requires not only the aspects validated in this experiment but also the setup and preparation associated with the device.

Finally, in Japan, 43.9% of older individuals aged ≥ 70 years prefer to receive care at home, compared to 34.6% of individuals in their 20s who desire home care^37^. Consequently, younger individuals may not readily embrace informal caregiving compared to older individuals, which limits the generalizability of the present study findings. However, in reality, 57.7% of care recipients receive care mainly from family members who either live with them or separately (informal caregivers)^34^, and challenges such as back pain and caregiving leave exist. Therefore, it is necessary to continue research not only on support for informal caregivers but also on expanding and improving formal caregiving services.

## Conclusion

We developed a caregiver-assistive device that can change and maintain the care recipient’s body in the lateral position. The study evaluated the feasibility of the device and subjective assessment of device-assisted care in comparison with those of the manual method by non-specialists. After a short period of practice, non-specialists were able to safely use the device well, and the care evaluations rated by the caregivers and care recipients were also higher than those of manual care. The study results suggest that informal caregivers can provide safe and comfortable care that is less burdensome than manual care by using a caregiver-assistive device.

## Data Availability

The datasets used and/or analyzed during the current study are available from the corresponding author upon request.

## References

[1] Organisation for Economic Co-Operation and Development. *Elderly Population (Indicator).* http://data.oecd.org/pop/elderly-population.htm (2023).

[2] Japan Cabinet. Office. *Annual Report on the Ageing Society [Summary] FY2021*. https://www8.cao.go.jp/kourei/english/annualreport/2021/pdf/2021.pdf (2021).

[3] Ministry of Health, Labour and Welfare. *Community-Based Integrated Care Systems.* https://www.mhlw.go.jp/stf/seisakunitsuite/bunya/hukushi_kaigo/kaigo_koureisha/chiiki-houkatsu/ (2023).

[4] Ministry of Internal Affairs and Communications Statistics Bureau. *Survey on Time Use and Leisure Activities 2021* 6–7. https://www.stat.go.jp/data/shakai/2021/pdf/gaiyoua.pdf (2021).

[5] Japan Cabinet Office in *Annual Report on the Ageing Society. Chapter 1 (2): Situation on Ageing Population.* https://www8.cao.go.jp/kourei/whitepaper/w-2022/html/zenbun/s1_1_2.html (2022).

[6] Llamas-Ramos R, Barrero-Santiago L, Llamas-Ramos I, Montero-Cuadrado F (2022). Effects of a family caregiver care programme in musculoskeletal pain and disability in the shoulder-neck region—A randomised clinical trial. Int. J. Environ. Res. Public Health.

[7] Hiel L (2015). Providing personal informal care to older European adults: Should we care about the caregivers' health?. Prev. Med..

[8] Jang SN, Avendano M, Kawachi I (2012). Informal caregiving patterns in Korea and European countries: A cross-national comparison. Asian Nurs. Res..

[9] Abdullahi A (2022). Prevalence of chronic non-specific low back pain among caregivers of stroke survivors in Kano, Nigeria and factors associated with it: A cross-sectional study. Front. Neurol..

[10] Jacob L (2020). Informal caregiving, chronic physical conditions, and physical multimorbidity in 48 low- and middle-income countries. J. Gerontol. A Biol. Sci. Med. Sci..

[11] Peters DH (2008). Poverty and access to health care in developing countries. Ann. N. Y. Acad. Sci..

[12] Mittelman MS, Haley WE, Clay OJ, Roth DL (2006). Improving caregiver well-being delays nursing home placement of patients with Alzheimer disease. Neurology.

[13] Litzelman K, Kent EE, Mollica M, Rowland JH (2016). How does caregiver well-being relate to perceived quality of care in patients with cancer? Exploring associations and pathways. J. Clin. Oncol..

[14] Darragh AR (2015). Musculoskeletal discomfort, physical demand, and caregiving activities in informal caregivers. J. Appl. Gerontol..

[15] Suzuki K, Tamakoshi K, Sakakibara H (2016). Caregiving activities closely associated with the development of low-back pain among female family caregivers. J. Clin. Nurs..

[16] Hignett S, Edmunds Otter M, Keen C (2016). Safety risks associated with physical interactions between patients and caregivers during treatment and care delivery in Home Care settings: A systematic review. Int. J. Nurs. Stud..

[17] Choi SD, Brings K (2015). Work-related musculoskeletal risks associated with nurses and nursing assistants handling overweight and obese patients: A literature review. Work.

[18] Dutta T, Holliday PJ, Gorski SM, Baharvandy MS, Fernie GR (2011). The effects of caregiver experience on low back loads during floor and overhead lift maneuvering activities. Int. J. Ind. Ergon..

[19] Omura Y (2019). Evaluation of the effectiveness of the sliding sheet in repositioning care in terms of working time and subjective fatigue: A comparative study with an experimental design. Int. J. Nurs. Stud..

[20] Amini Pay N, Sommerich CM, Lavender SA (2021). Assessment of alternative methods for informal caregivers to perform patient repositioning tasks. Appl. Ergon..

[21] Omura Y, Hirata M, Yoshimine T, Nakatani E, Inoue T (2022). Development and evaluation of a new assistive device for low back load reduction in caregivers: An experimental study. Sci. Rep..

[22] Tateishi K, Matsubayashi T, Yoshimoto K, Sakemi T (2013). An investigation of the basic education of Japanese nurses: Comparison of competency with European nurses. Nurse Educ. Today.

[23] Ministry of Internal Affairs and Communications Statistics Bureau (e-stat). *National Health and Nutrition Survey 2018*. https://www.e-stat.go.jp/dbview?sid=0003224177 (2018).

[24] Omura Y, Yamagami Y, Inoue T (2019). Evaluation of repositioning and turning care provided to the general elderly people and recognition of assistive device use. J. Jpn. Health Med. Assoc..

[25] Perry AG, Potter PA, Ostendorf WR, Laplante N (2021). Clinical Nursing Skills and Techniques.

[26] Wensley C, Botti M, McKillop A, Merry AF (2017). A framework of comfort for practice: An integrative review identifying the multiple influences on patients' experience of comfort in healthcare settings. Int. J. Qual. Health Care.

[27] Japanese Society of Nursing Science Nursing Academic Terminology Review Committee. *JANSpedia—A Glossary of Important Terms That Make Up Nursing Science*. https://scientific-nursing-terminology.org/ (2023)

[28] Borg GAV (1982). Psychophysical bases of perceived exertion. Med. Sci. Sports Exerc..

[29] Borg GAV (1970). Perceived exertion as an indicator of somatic stress. Scand. J. Rehabil. Med..

[30] Salmani Nodooshan H, Choobineh A, Razeghi M, Shahnazar Nezhad Khales T (2017). Designing, prototype making and evaluating a mechanical aid device for patient transfer between bed and stretcher. Int. J. Occup. Saf. Ergon..

[31] Hwang J, Kuppam VA, Chodraju SSR, Chen J, Kim JH (2019). Commercially available friction-reducing patient-transfer devices reduce biomechanical stresses on caregivers' upper extremities and low back. Hum. Factors.

[32] Akino S, Hinotsu A, Muramatsu M (2018). Differences in use of hands by nurses and nursing students: Force exerted by the fingers and palms on the area of contact in postural change. J. Jpn. Soc. Nurs. Res..

[33] Huang W, He B, Wang YH, Ma WJ, Zhou J (2020). Disability severity and home-based care quality in older adults: The mediating effects of social support and caregiver competence. J. Nurs. Res..

[34] Ministry of Health, Labour and Welfare. *Comprehensive Survey of Living Conditions 2022. Chapter IV: Situation on Long-Term Care.* https://www.mhlw.go.jp/toukei/saikin/hw/k-tyosa/k-tyosa22/index.html (2022).

[35] Dahlhamer J (2018). Prevalence of chronic pain and high-impact chronic pain among adults—United States, 2016. Morb. Mortal. Wkly. Rep..

[36] Hayase D (2004). Age-related changes in activities of daily living ability. Austral. Occup. Ther. J..

[37] Cabinet Office. *Public Opinion Survey Regarding Nursing Care Insurance System 2. Home Care and Facility Care.* https://survey.gov-online.go.jp/h22/h22-kaigohoken/2-2.html (2010).

